# Ectropion and Conjunctival Mass in a Patient with Primary Bilateral Conjunctival Amyloidosis

**DOI:** 10.1155/2016/5610753

**Published:** 2016-11-16

**Authors:** Alessandro Meduri, Miguel Rechichi, Cosimo Mazzotta, Sergio Zaccharia Scalinci, Mahmoud O. Jaroudi

**Affiliations:** ^1^Department of Ophthalmology, University of Messina, Messina, Italy; ^2^Department of Ophthalmology, University of Magna Graecia, Catanzaro, Italy; ^3^Department of Medical and Surgical Neurosciences, Ophthalmology Unit, Cornea Center, Siena University Hospital, Siena, Italy; ^4^Low Vision Center, University of Bologna, Bologna, Italy; ^5^Department of Ophthalmology, American University of Beirut Medical Center, Beirut, Lebanon

## Abstract

*Background.* Amyloidosis is a group of disorders characterized by deposition of an extracellular protein, known as amyloid, in an abnormal fibrillar form with highly characteristic histopathologic staining properties. The clinical presentation can vary from a focal, localized lesion where amyloidosis has minor clinical consequences to extensive systemic disease that can involve any organ system of the body. Ocular amyloidosis can occur as a localized lesion or as a part of a systemic disorder. Conjunctival amyloidosis is an uncommon condition that is rarely associated with systemic disease. It may be a manifestation of an immunologic disorder.* Case Report.* We report the case of a patient with bilateral conjunctival amyloidosis who was referred to us with the suspicion of a malignant conjunctival lesion. Examination of both eyes showed a yellow-pink mass with prominent intrinsic vessels, subconjunctival hemorrhage, and ectropion of the left lower eyelid. Diagnosis of primary localized conjunctival amyloidosis was made based on histopathologic evaluation of incisional biopsy and negative systemic work-up.* Conclusion.* Ocular amyloidosis is a rare disease that is slowly progressive and has a wide variety of clinical presentations. Consequently, the clinical diagnosis is often overlooked or delayed. Definitive diagnosis is achieved through histopathologic evaluation of biopsy specimen.

## 1. Background

Amyloidosis is a group of disorders characterized by the deposition of an insoluble extracellular protein, known as amyloid, in an abnormal fibrillar form with highly characteristic histopathologic staining properties [1].

Named by Virchow in 1854 on the basis of its color after staining with iodine and sulfuric acid, amyloid fibrils share an identical secondary structure, the *β*-pleated sheet conformation, and a unique ultrastructure. All amyloid deposits contain the pentraxin serum amyloid P (SAP) and glycosaminoglycans [2]. Its ultrastructural configuration consists of 10–15 nm diameter stiff fibrils composed of diverse proteins [3]. Amyloid is an eosinophilic, amorphous protein that has been reported to deposit in virtually any tissue or organ and when extensive may attain tumourous proportions [4]. Localized deposition of amyloid may occur in individual organs in the absence of systemic involvement. The reason for localized deposition is unknown; it is hypothesized that deposits result from local synthesis of amyloid protein or from plasmatic origin by deposition of light chains produced elsewhere [5].

Generally, there are two clinical presentations of conjunctival amyloidosis: a focal, localized lesion with minor clinical consequences or a lesion associated with extensive systemic disease that can involve almost any organ system of the body, leading to severe pathophysiologic damage [6]. Primary localized conjunctival amyloidosis is a very rare disease. In a review of 2,455 conjunctival lesions submitted to a pathology laboratory, only five patients (0.002%) were found to have conjunctival amyloidosis [7]. Definitive diagnosis consists of histopathologic evaluation of biopsy showing amyloid material in the conjunctival tissue. Systemic evaluation should be carried out in order to rule out the presence of systemic amyloidosis [8].

We herein report the rare case of a patient with primary bilateral conjunctival amyloidosis who was initially misdiagnosed.

## 2. Case Report

A 55-year-old white woman presented with a 7-month history of hypertrophic conjunctivitis in both eyes. She was treated elsewhere with topical therapy, but persistence of signs and symptoms prompted referral to our center for suspicion of ocular lymphoma. She had history of allergic conjunctivitis in the right eye diagnosed histologically 15 years ago. Her past medical and social history were noncontributory. There was no history of previous ocular surgeries.

**Figure 1:**
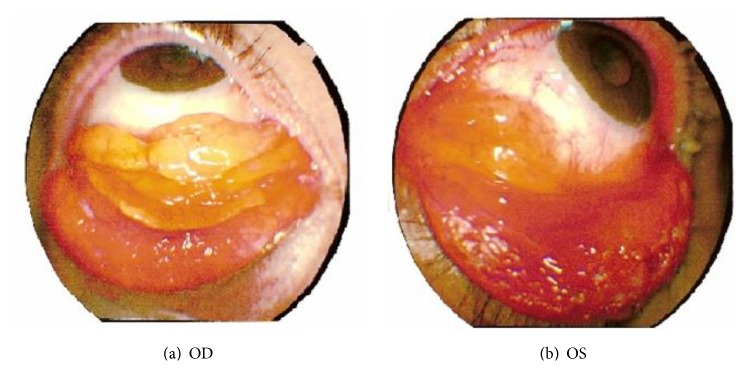
External photos of right (a) and left (b) eye showing bilateral yellow-pink conjunctival mass in the lower fornices with subconjunctival hemorrhage and left eye ectropion.

Visual acuity on presentation was 6/6 in each eye. Intraocular pressure measured 13 mmHg bilaterally. Anterior segment examination of both eyes showed papillary hypertrophy of the palpebral conjunctivae and a fleshy yellow-pink mass with prominent intrinsic vessels within the lower fornices associated with subconjunctival hemorrhage. In addition, ectropion and lower eyelid epithelium metaplasia were seen in the left eye (Figure 1). Fundus examination was normal bilaterally. CT scan showed no orbital extension.

**Figure 2:**
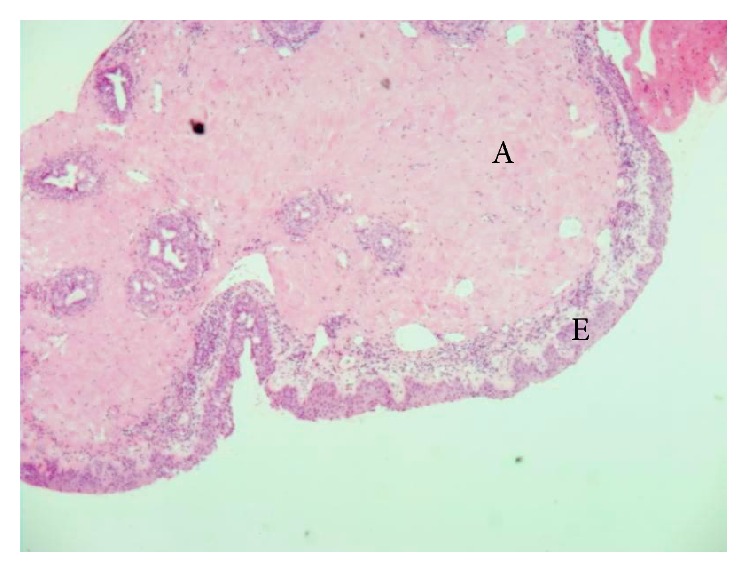
Photomicrograph of the biopsy specimen shows deposit of amyloid in the conjunctival chorion stained in pink (Congo red 50x); E = conjunctival epithelium and A = amyloid.

**Figure 3:**
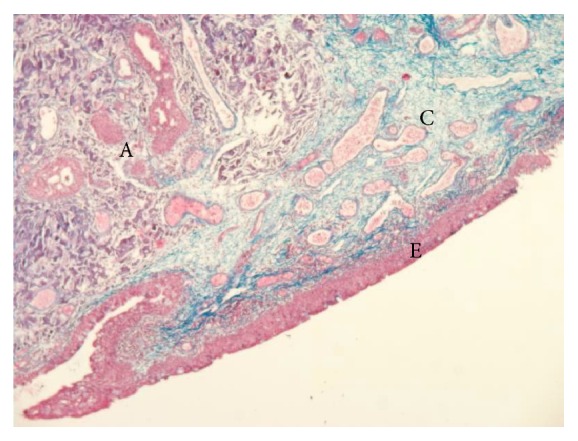
Photomicrograph of the biopsy specimen shows deposit of amyloid stained in gray- violet and collagen in blue (Mallory's triple stain 50x). Conjunctival chorion shows waxy infiltration. Epithelium is not involved. E = conjunctival epithelium, A = amyloid, and C = connective tissue.

Excisional biopsy for histopathologic evaluation followed by cryotherapy was performed. The mass was composed of fibrous tissue bordered by parakeratosis malpighian epithelium with papillomatosis and contiguous typical conjunctival mucosa with nonspecific chronic inflammatory reaction. Histopathologic examination revealed acellular amorphous eosinophilic material that was congophilic and showed apple-green birefringence and dichroism with polarization microscopy, consistent with amyloidosis (Figures 2 and 3).

A thorough systemic review and investigations by an internist targeted at establishing secondary amyloidosis were all negative. In particular, the liver function tests, serum immunoglobulin levels, bone marrow aspiration, and serum and urine electrophoresis for Bence-Jones proteins were all normal. The work-up to rule out systemic involvement includes the following:

Complete blood count
Urine analysis
Erythrocyte sedimentation rate
Serum electrolytes
Serum immunoglobulin levels
Serum protein immunoelectrophoresis
Urine protein immunoelectrophoresis
Urine Bence-Jones protein levels
Serum immune complex levels
Serum T protein levels
Prothrombin time and activated partial prothrombin time
Liver function tests
Allergological test
Echocardiogram
Electrocardiogram
Abdomen TC
Abdomen ultrasonography
Bone marrow aspiration biopsy
Subcutaneous fat aspirate

After establishing the diagnosis of a localized conjunctival amyloidosis, the patient underwent surgical debulking of the amyloid tumor in both eyes in addition to left ectropion repair. On her last follow-up examination one year after the surgery, the patient had normal anterior segment findings with no clinical evidence of recurrence.

## 3. Discussion

We presented a rare case of localized bilateral conjunctival amyloidosis in the absence of systemic involvement. According to a new classification, amyloidosis is categorized as localized or systemic [9, 10].

Systemic amyloidosis can be either hereditary or acquired. The most common form of local amyloidosis is caused by the local deposition of monoclonal immunoglobulin light chains by a usually benign B-cell or plasma-cell clone and is called localized amyloid light chain amyloidosis [9, 11, 12].

Some types of amyloidosis are associated with cardiac, cerebral, or renal involvement, and so all cases of periocular and orbital amyloidosis are investigated for systemic involvement, although this is rare.

Localized conjunctival amyloidosis is a benign ocular surface disease that occurs within the upper fornix more commonly than the lower fornix and is more commonly unilateral. Its morphologic appearance on clinical examination may mimic other more serious or malignant diseases such as lymphoma. Conjunctival deposits are usually painless but may gradually grow in size causing a significant local swelling and irritation. Recurrent subconjunctival hemorrhages have been reported to be associated with conjunctival amyloidosis and it is believed to be due to increased fragility of orbital vasculature secondary to amyloid deposits [13].

Ocular amyloidosis can involve different parts of the globe and/or orbit causing a wide range of signs and symptoms based on its location. This may often lead to delayed or missed diagnosis. The diagnosis of conjunctival amyloidosis in our patient was delayed for 7 months. In the largest reported series of periocular and orbital amyloidosis, Leibovitch et al. have shown that the most common presenting sign in their group of patients was a mass formation or tissue infiltration (23 out of 24 patients) followed by ptosis (13/24 patients). Nineteen (79%) of their cases were unilateral and 5 were bilateral. Concerning anatomical location of amyloid deposits, 13 patients (56.2%) had only conjunctival involvement, 7 patients (30.4%) had a periocular mass but no obvious conjunctival involvement, and 3 patients (13%) had both conjunctival and periocular tissue involvement [14]. Our patient had bilateral conjunctival involvement, which seems to be the most common site of ocular involvement, leading to a protruding mass within the lower fornix. The location of the tumor within the lower fornix leads to left ectropion.

The rarity of the disease and its diverse clinical presentations pose a diagnostic challenge to the examining ophthalmologist. The most common reported signs and symptoms include visible or palpable periocular mass or tissue infiltration and ptosis. Other reported signs and symptoms include pain or periocular discomfort, recurrent periocular subcutaneous hemorrhages, ocular motility disturbances, proptosis, diplopia, and globe displacement [13, 14]. The clinical picture and morphologic appearance alone are not enough to make a diagnosis of conjunctival amyloidosis. A definitive diagnosis is established by histopathologic examination of biopsy specimens with specific stains showing a pathognomonic red-green dichroism when preparations stained with Congo red are viewed in intense unidirectional polarized light [9]. Amyloid fibrillary protein was found to infiltrate and deposit also in vessel walls. These deposits increase the rigidity and disrupt the integrity of conjunctival vessels predisposing to spontaneous subconjunctival hemorrhage.

Any case of conjunctival or ocular amyloidosis should be extensively worked up to rule out systemic involvement, although uncommon. In their largest case series, Leibovitch et al. reported that only one patient was confirmed to have systemic amyloidosis involving the mouth, stomach, and duodenum [14]. Our patient was worked up thoroughly by an internist and there was no evidence of systemic involvement. Although the vast majority of patients with primary conjunctival deposits have no positive systemic findings, progression of a local primary amyloidosis to a systemic disease has been reported.

Treatment of ocular amyloidosis can be complex and challenging depending on the location, extent of ocular involvement, associated systemic involvement, and patient's general health. Different treatment modalities for localized amyloidosis have been described. These include surgical debulking, radiotherapy, liquid nitrogen cryotherapy, and observation. Surgical debulking remains the mainstay of treatments in patients with symptomatic diseases including ocular motility disturbances, compressive optic neuropathy, and disfiguring cosmetic appearance. Observation is a choice of treatment in asymptomatic patients with localized amyloidosis. In patients with medical contraindications for surgery or with extensive infiltrative disease where surgical treatment might be very difficult, radiotherapy and supportive treatment may be useful [14–17].

In case ocular amyloidosis is secondary to a systemic disease, the systemic disease should be addressed and treated first.

Our patient had a localized bilateral conjunctival amyloidosis with no orbital or intraocular involvement. She underwent bilateral surgical excision with repair of left lower ectropion. No recurrence was observed on her last follow-up one year after surgery.

## 4. Conclusion

Ocular amyloidosis is a rare disease that is slowly progressive and has a wide variety of clinical presentations. Consequently, the clinical diagnosis is often overlooked or delayed. Definitive diagnosis is achieved through histopathologic evaluation of biopsy specimen. Although the majority of cases occur as a primary localized disease, intensive systemic work-up should be carried out to rule out systemic involvement. Choice of treatment should be individualized taking into consideration the extent of the disease, presence of systemic involvement, and the patient's general health.
